# Comparison of Conventional Keratometry and Total Keratometry in Normal Eyes

**DOI:** 10.1155/2020/8075924

**Published:** 2020-04-13

**Authors:** Rie Hoshikawa, Kazutaka Kamiya, Fusako Fujimura, Nobuyuki Shoji

**Affiliations:** ^1^Department of Rehabilitation, Orthoptics and Visual Science Course, School of Allied Health Science, Kitasato University, Sagamihara, Japan; ^2^Department of Ophthalmology, School of Medicine, Kitasato University, Sagamihara, Japan

## Abstract

**Purpose:**

The relationship between conventional keratometry and total keratometry has not been fully investigated. This study was aimed at conventional keratometry measured with the automated keratometer and total keratometry with the corneal tomographer in ophthalmologically normal subjects.

**Methods:**

We enrolled fifty eyes of 50 consecutive subjects (mean age ± standard deviation, 34.9 ± 8.0 years) who have no ophthalmologic diseases, other than refractive errors, with no history of ocular surgery. Conventional keratometry was measured with the automated keratometer. The total keratometry, the true net power (TNP), and the total corneal refractive power (TCRP) were measured with the Scheimpflug camera, and the real power (RP) was measured with anterior segment optical coherence tomography (As-OCT). Anterior keratometries (Km and AvgK) were also measured with the Scheimpflug camera and the As-OCT, respectively.

**Results:**

Conventional keratometry was 43.64 ± 1.48 D, which was significantly higher than the TCRP (42.94 ± 1.45 D, *p* = 0.042), the TNP (42.13 ± 1.37 D, *p* < 0.001), and the RP (42.62 ± 1.39 D, *p* = 0.001, Dunnett's test). We found significant correlations between conventional keratometry and each total corneal power (the TCRP (Pearson's correlation coefficient *r* = 0.986, *p* < 0.001), the TNP (*r* = 0.986, *p* < 0.001), the RP (*r* = 0.987, *p* < 0.001), the Km (*r* = 0.990, *p* < 0.001), and the AvgK (*r* = 0.991, *p* < 0.001)). The intraclass correlations of conventional keratometry with the TCRP, the TNP, the RP, the Km, and the AvgK were 0.986, 0.983, 0.985, 0.990, and 0.990, respectively. We found no significant differences in the keratometric data measured with the automated keratometer, the Scheimpflug camera, and the As-OCT (ANOVA, *p* = 0.729).

**Conclusions:**

Conventional keratometry was significantly larger than total keratometry, by approximately 0.70 to 1.52 D, in ophthalmologically normal subjects. By contrast, there were no significant differences in the keratometric data among the three devices. It is suggested that conventional keratometry overestimates the total corneal power in daily practice.

## 1. Introduction

Conventional keratometry is mostly used not only for corneal power measurements but also for intraocular lens (IOL) power calculation, in daily practice. It is theoretically calculated by the anterior corneal curvature and the standard refractive index of 1.3375 to estimate the total corneal power. Recent advances in diagnostic devices in ophthalmology have allowed us to directly obtain the total corneal power by the measurements of the anterior and posterior corneal surfaces. It is possible that the differences between conventional keratometry and total keratometry might become larger in some eyes, since the former keratometry measurement was calculated based on the assumption that the anterior-posterior corneal radius ratio remains constant.

There have so far been several studies comparing simulated keratometry and total keratometry, mostly based on the corneal tomographer, such as the Scheimpflug camera or the anterior segment optical coherence tomography (As-OCT) [1–4]. However, to the best of our knowledge, the relationship between conventional keratometry, which is mostly used in daily practice, and total keratometry has not been fully elucidated. It may give us intrinsic insights in the biometric differences between these two keratometries and in the determination of the accurate corneal refractive power. The goal of the current study is twofold: to compare conventional keratometry measured with the automated keratometer with total keratometry measured with the Scheimpflug camera as well as the As-OCT and to evaluate the correlation between conventional keratometry and total keratometry in an ophthalmologically normal population.

## 2. Materials and Methods

### 2.1. Study Population

This study protocol was registered with the University Hospital Medical Information Network Clinical Trial Registry (000037929). We retrospectively reviewed the biometric data of fifty eyes of 50 subjects with normal corneal and ocular findings applying for a contact lens fitting or a refractive surgery consultation at Kitasato University Hospital. The subjects were enrolled in a continuous cohort. Keratoconus and pellucid marginal degeneration cases were excluded from this study.

### 2.2. Corneal Power Measurements

We performed corneal power measurements using three instruments (the autokeratometer, the Scheimpflug camera, and the As-OCT) in a random fashion in these subjects.

Conventional keratometry was determined using an automated keratometer (TONOREF II, Nidek, Gamagori, Aichi, Japan), which was calculated by measuring the anterior corneal curvature and using the standard keratometric index of 1.3375.

As the total keratometry, we obtained the true net power (TNP) and the total corneal refractive power (TCRP) using the Scheimpflug camera (Pentacam HR, OCULUS, GmbH, Wetzlar, Germany) and the real power (RP) using the As-OCT (CASIA 2, Tomey, Nagoya, Aichi, Japan). We checked image quality for each eye, and only one examination with a high-quality factor was documented.

The TNP and the TCRP on the central 15° ring (equal to the 3.0 mm ring) around the corneal apex were automatically measured with the Scheimpflug camera. The TNP was calculated based on the Gaussian optic formula. The TCRP was calculated based on ray tracing through the anterior and posterior corneal surfaces according to Snell's law.

The RP on the 3.0 mm ring around the corneal apex was automatically measured with the As-OCT. The RP was calculated as the sum of the anterior and posterior corneal powers adjusted by the corneal thickness based on the Gaussian optic formula.

Anterior keratometries (Km, cornea front; AvgK, keratometric), by measuring the anterior corneal curvature and using the standard keratometric index of 1.3375, were also obtained with the Scheimpflug camera and the As-OCT, respectively, in order to compare the keratometric data among the three devices.

To assess the effect of the posterior corneal power on the differences between conventional keratometry and total keratometry, the posterior corneal power on the 3.0 mm ring was also measured with the Scheimpflug camera and the As-OCT.

This retrospective review of the clinical charts was approved by the Institutional Review Board of Kitasato University Hospital (B18-0254) and followed the tenets of the Declaration of Helsinki. Our Institutional Review Board waived the requirement for informed consent for this retrospective study. The data that support the findings of the present study are available from the corresponding author upon reasonable request.

### 2.3. Statistical Analysis

All statistical analyses were conducted using a statistical software (Bell Curve for Excel, Social Survey Research Information Co., Ltd., Tokyo, Japan). Since all data fulfilled the criteria for normal distribution by the Kolmogorov-Smirnov test, Pearson's correlation coefficient was calculated to assess the relationship between the two variables. One-way analysis of variance (ANOVA) was used to compare conventional keratometry and total keratometry, with Dunnett's test being employed for multiple comparison. The results are expressed as mean ± standard deviation, and a value of *p* < 0.05 was considered statistically significant.

## 3. Results

**Figure 1:**
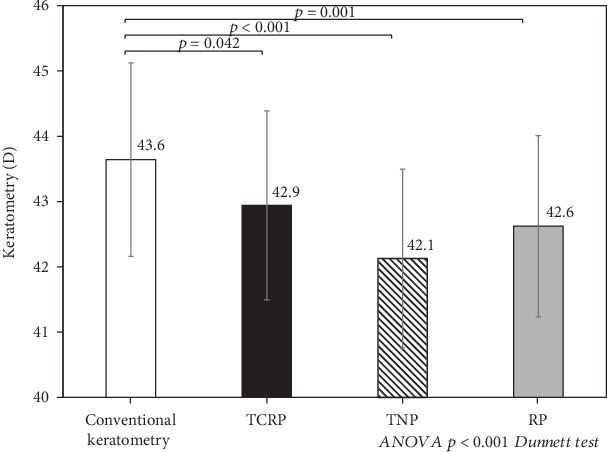
A graph showing the conventional keratometry and total keratometry data (TCRP, TNP, and RP).

Table 1 shows the demographics of the study population.  Figure 1 shows conventional keratometry, TCRP, TNP, and RP data. There were significant differences among these data (ANOVA, *p* < 0.001). Conventional keratometry was significantly higher than the TCRP (*p* = 0.042), the TNP (*p* < 0.001), and the RP (*p* = 0.001, Dunnett's test). Conventional keratometry overestimated the TCRP, the TNP, and the RP, by 0.70 ± 0.24 diopter (D) (95% confidence interval (CI), 0.22 to 1.18 D), 1.52 ± 0.26 D (1.01 to 2.03 D), and 1.02 ± 0.25 D (0.54 to 1.50 D), respectively.

**Figure 2:**
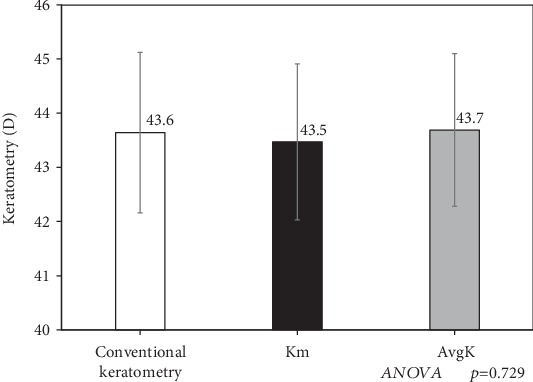
A graph showing the keratometric data measure with the automated keratometer (conventional keratometry), the Scheimpflug camera (Km), and the anterior segment optical coherence tomography (AvgK).

Figure 2 shows the keratometric data measured with the automated keratometer, the Scheimpflug camera, and the As-OCT. There were no significant differences in the keratometric data among the three devices (ANOVA, *p* = 0.729).

**Figure 3:**
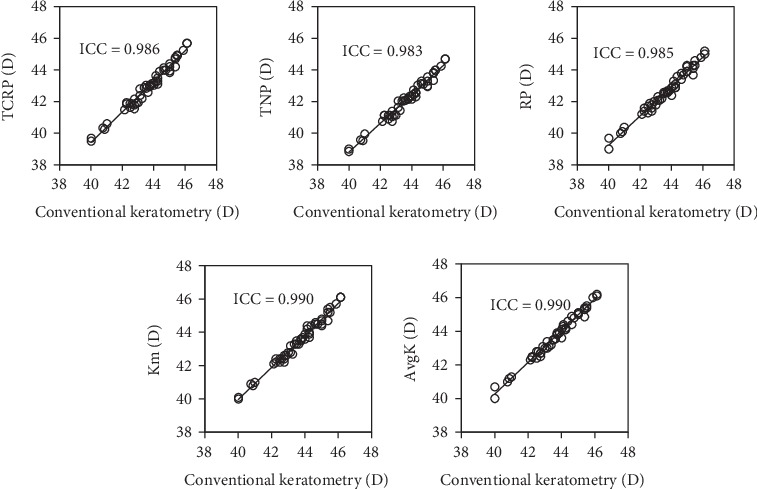
Graphs showing significant associations and the intraclass correlations (ICCs) between conventional keratometry and total keratometry (TCRP, TNP, and RP) or anterior keratometry (Km and AvgK).

Figure 3 shows the relationship of conventional keratometry with the TCRP (Pearson's correlation coefficient *r* = 0.986, *p* < 0.001), the TNP (*r* = 0.986, *p* < 0.001), the RP (*r* = 0.987, *p* < 0.001), the Km (*r* = 0.990, *p* < 0.001), and the AvgK (*r* = 0.991, *p* < 0.001). The intraclass correlations of conventional keratometry with the TCRP, the TNP, the RP, the Km, and the AvgK were 0.986, 0.983, 0.985, 0.990, and 0.990, respectively.

**Figure 4:**
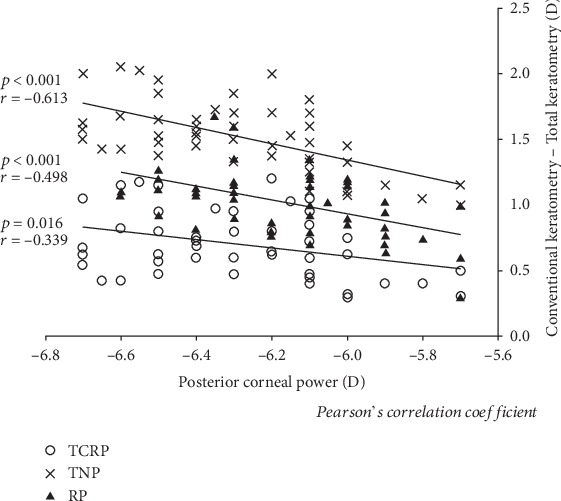
A graph showing significant associations between the posterior corneal power and the differences between conventional keratometry and total keratometry (TCRP: Pearson's correlation coefficient *r* = −0.339, *p* = 0.016; TNP: *r* = −0.613, *p* < 0.001; and RP: *r* = −0.498, *p* < 0.001).

Figure 4 shows the relationship between the posterior corneal power and the differences between conventional keratometry and total keratometry. We found significant correlations between the posterior corneal power and the differences of conventional keratometry with the TCRP (Pearson's correlation coefficient *r* = −0.339, *p* = 0.016), the TNP (*r* = −0.613, *p* < 0.001), and the RP (*r* = −0.498, *p* < 0.001).

## 4. Discussion

In the current study, our results showed that there were significant associations between conventional keratometry and total keratometry but that conventional keratometry was significantly higher than total keratometry in an ophthalmologically normal population. These results imply that conventional keratometry may overestimate the total corneal power even in healthy subjects in daily practice. We believe that the differences in the two keratometries were not clinically negligible, since the mean differences reached approximately 0.70 to 1.52 D. On the other hand, our results showed that there were no significant differences in the keratometric data measured with the automated keratometer, the Scheimpflug camera, and the As-OCT. These results suggest that the keratometric data, by measuring the anterior corneal curvature and using the standard keratometric index of 1.3375, were not significantly different among these clinical devices.

Table 2 shows the summary of previous studies comparing anterior keratometry and total keratometry [1–5]. Our findings were in line with previous studies, in that anterior keratometry was significantly higher than total keratometry. However, most studies have merely focused on a comparison between simulated keratometry and total keratometry, measured with the same corneal tomographer [1, 3, 4]. There has only been one preliminary study comparing conventional keratometry and total keratometry. Olsen and Jeppesen demonstrated that conventional keratometry was higher than the ray-traced corneal power by 1.02 ± 0.50 D and that the difference between the ray-traced corneal power and the TCRP was 0.03 ± 0.25 D [5]. However, they primarily focused on the ray-traced corneal power obtained by the Scheimpflug camera, and the small sample size (*n* = 20) might be insufficient for drawing scientific conclusions.

Conventional keratometry is most commonly used for corneal power measurements for IOL power calculation in a clinical setting. It is theoretically calculated as the estimated total keratometry by measuring the anterior corneal curvature and using a standard refractive index of 1.3375. However, the standard refractive index is determined only by the assumption that the corneal curvature of 7.5 mm is equal to the corneal power of 45 D [6, 7]. Indeed, it has been demonstrated that the actual refractive index of the cornea was 1.329 [8], 1.3273 [9], and 1.3281 [10], all of which were smaller than the standard refractive index of 1.3375. Ho et al. [10] showed that the use of an actual refractive index of 1.3281 reflected more accurately the total corneal power than that of 1.3375.

As shown in Figure 4, we found a significant negative correlation between the posterior corneal power and the differences between conventional keratometry and total keratometry. The keratometric differences tended to become lager, when the posterior corneal power decreased, which was in accordance with previous findings by Camps et al. [11], using the Gaussian equation, that the difference between the keratometric power and the Gaussian corneal power was theoretically increased, when the posterior corneal curvature was steepened. It is suggested that conventional keratometry overestimates the total cornea power, especially in the eyes with a steep posterior corneal curvature.

The percentage of refractive error contribution of corneal power measurements was estimated to be approximately 8% [12], which was not clinically negligible, since modern cataract surgery is deemed as one of refractive surgeries. Accordingly, precise total keratometry, instead of conventional keratometry, should be utilized for IOL power calculation. There have been so far several studies on the predictability of IOL power calculation, using the total corneal power [2, 3, 13], but the benefits of the use of the total corneal power were still limited, possibly because most IOL power calculation formulas have been already optimized for clinical use. For example, assuming a standard axial length of 24.0 mm, a standard IOL power of 20.0 D (*A* constant 119.0), and the use of the SRK/T formula, the use of the conventional keratometry (43.64 D), the TCRP (42.94 D), the TNP (42.13 D), the RP (42.62 D), the Km (43.47 D), and the AvgK (43.69 D) will result in the predicted refraction of -0.27 D, 0.24 D, 0.83 D, 0.47 D, -0.14 D, and -0.30 D, respectively. We should be aware that clinical optimization is still necessary when using total keratometry for IOL power calculation, even if it may reflect the actual corneal power.

There are at least two limitations to this study. First, the study was performed in a retrospective fashion. Second, we investigated keratometric data in subjects for a contact lens fitting or a refractive surgery consultation, and thus, it might be biased. A further prospective study with another normal population is still necessary to confirm our findings.

In conclusion, we compared conventional keratometry measured with the autokeratometer with total keratometry measured with the Scheimpflug camera and the As-OCT in healthy subjects. Our findings showed that there were significant associations between conventional keratometry and total keratometry but that conventional keratometry was significantly higher than total keratometry. By contrast, our findings showed that there were no significant differences in the keratometric data measured with the automated keratometer, the Scheimpflug camera, and the As-OCT. It is indicated that conventional keratometry overestimates the total corneal power by approximately 0.70 to 1.52D. Our findings may be helpful for understanding the fundamental differences in the two keratometries in a clinical setting.

## Figures and Tables

**Table 1:** Demographics of the study population.

Characteristics	
Number of subjects	50
Age	34.9 ± 8.0 years (95% CI, 19.2 to 50.5 years)
Gender	Male : female = 24 : 26
Conventional keratometry	43.64 ± 1.48 D (95% CI, 40.74 to 46.55 D)
Km, cornea front	43.47 ± 1.44 D (95% CI, 40.65 to 46.29 D)
AvgK, keratometric	43.69 ± 1.41 D (95% CI, 40.93 to 46.45 D)
Posterior corneal power	Pentacam: -6.29 ± 0.26 D (95% CI, -6.79 to -5.78 D) CASIA 2: -6.17 ± 0.23 D (95% CI, -6.63 to -5.71 D)
Total corneal refractive power (TCRP)	42.94 ± 1.45 D (95% CI, 40.10 to 45.78 D)
True net power (TNP)	42.13 ± 1.37 D (95% CI, 39.44 to 44.81 D)
Real power (RP)	42.62 ± 1.39 D (95% CI, 39.90 to 45.35 D)

The results are expressed as mean ± standard deviation (95% CI = confidence interval). D = diopter.

**Table 2:** Summary of previous studies on comparing simulated keratometry or conventional keratometry, and total keratometry.

Author	Year	*N*	Device		Differences between simulated keratometry and total keratometry (D)
			Simulated keratometry (conventional keratometry)	Total keratometry	
Wang et al. [1]	2011	94	Galilei (Ziemer, Switzerland)		GEP: 1.30 D^∗^
Savini et al. [2]	2013	41	Keraton (Optikon, Spain)	Pentacam HR (OCULUS, Germany)	TNP: 1.30 D
Savini et al. [3]	2017	114	Sirius (CSO Florence, Italy)		TCP: 0.56 ± 0.23 D^§^
Hasegawa et al. [4]	2018	501	CASIA (Tomey, Japan)		RP: 1.08 ± 0.12 D
Olsen and Jeppesen [5]	2018	20	ARK700^†^ (conventional keratometry) (Nidek, Japan)	Pentacam HR (OCULUS, Germany)	Ray-traced corneal power: 1.02 ± 0.50 D
Current study		50	TONOREF II^†^ (conventional keratometry) (Nidek, Japan)	Pentacam HR (OCULUS, Germany) CASIA 2 (Tomey, Japan)	TCRP: 0.70 ± 0.24 D TNP: 1.52 ± 0.26 D RP: 1.02 ± 0.25 D

Simulated keratometry was measured with the corneal topography/tomography. ^†^Conventional keratometry was measured with the automated keratometer. ^∗^GEP = Gaussian equivalent power calculated by the Gaussian formula. ^§^TCP = total corneal power calculated by ray tracing.

## Data Availability

The data that support the findings of this study are available from the corresponding author, KK, upon reasonable request.
